# Prospective evidence for independent nitrogen and phosphorus limitation of grasshopper (*Chorthippus curtipennis*) growth in a tallgrass prairie

**DOI:** 10.1371/journal.pone.0177754

**Published:** 2017-05-16

**Authors:** Madison Rode, Nathan P. Lemoine, Melinda D. Smith

**Affiliations:** Department of Biology, Graduate Degree Program in Ecology, Colorado State University, Fort Collins, Colorado, United States of America; Natural Resources Canada, CANADA

## Abstract

Insect herbivores play a pivotal role in regulating plant production and community composition, and their role in terrestrial ecosystems is partly determined by their feeding behavior and performance among plants of differing nutritional quality. Historically, nitrogen (N) has been considered the primary limiting nutrient of herbivorous insects, but N is only one of many potential nutrients important to insect performance. Of these nutrients, phosphorus (P) is perhaps the most important because somatic growth depends upon P-rich ribosomal RNA. Yet relatively few studies have assessed the strength of P-limitation for terrestrial insects and even fewer have simultaneously manipulated both N and P to assess the relative strengths of N- and P-limitation. Here, we tested for potential N and P limitation, as well as N:P co-limitation, on *Chorthippis curtipennis* (Orthoptera, Acrididae), an abundant member of arthropod communities of central US prairies. Our results demonstrate weak evidence for both N and P limitation of *C*. *curtipennis* growth rates in laboratory feeding assays. Importantly, P-limitation was just as strong as N-limitation, but we found no evidence for NP co-limitation in our study. Furthermore, nutrient limitation was not apparent in field studies, suggesting that insect growth rates may be predominately controlled by other factors, including temperature and predation. Our results suggest that P should be jointly considered, along with N, as a primary determinant of herbivore feeding behavior under both current and future climate conditions.

## Introduction

Herbivores play a pivotal role in regulating plant production and community composition via both top-down [1] and bottom-up [2–4] mechanisms. The impacts of insect herbivores, in particular, often rival or exceed those of their vertebrate counterparts [5–7], and insects often regulate plant biomass [8,9], increase species diversity [10], determine the outcomes of competitive interactions [6,11,12], and enhance nutrient recycling rates [4,5,13]. Since these processes result from plant consumption by, and subsequent growth of, insect herbivores, it is important to understand the nutritional constraints on insect performance and their subsequent community-level impacts.

Historically, nitrogen (N) has been considered the primary limiting nutrient of herbivorous insects because insects possess 3–6 times higher N content in bodily tissues than is found in foliage [14–16]. This stoichiometric imbalance suggests that insects struggle to meet basic N requirements needed for protein synthesis when fed low N diets [17]. Consequently, insects feeding on N-enriched plants often experience higher growth [18–20], survival [21–24], and reproductive rates [22,25]. Such increased consumption, growth, and population sizes of insects often lead to greater control of plant biomass and community composition in N-enriched conditions [6,26]. Yet N is only one of many potential nutrients important to insect performance; grasshopper densities, for example, are correlated with multiple macro- and micro-nutrients that have received comparatively little attention in studies of insect nutrition [27].

Of these other nutrients, phosphorus (P) is perhaps the most important to insects for several reasons. First, similar to N, insects contain 2–5 times more P than do plant tissues [16], such that foliage P cannot meet an insect’s P requirements. Second, rapid growth rates, particularly during larval stages, increase the RNA:protein ratio in bodily tissues [28]. Since RNA is a P-rich molecule, this implies that any increase in growth rate requires increased P-intake. Indeed, high foliar P concentrations often stimulate insect growth rates [29–33], and also increases the probability of survival [34], fecundity [35], and population densities [36]. However, the positive benefits of P on insect performance are not universal; P-enrichment might provide no additional benefit or even become deleterious to insect performance if other nutrients are simultaneously limiting [15,34,37]. If, for example, insects experience both N- and P-limitation, then P-enrichment might have negligible effects on insect performance unless N-limitation is simultaneously alleviated [38]. Unfortunately, relatively few studies have experimentally tested for both N- and P-limitation of insect herbivores to determine whether P-limitation depends upon foliar N, *i*.*e*. the N:P ratio [38,39].

Here, we tested for potential N and P limitation, as well as N:P co-limitation, on *Chorthippus curtipennis* (Orthoptera, Acrididae), an abundant member of arthropod communities in central US prairies that is generally considered a grass specialist [40,41]. We tested four specific hypotheses: (1) *C*. *curtipennis* would be N-, but not P-limited. In this case, N-enrichment of foliage would yield more rapid growth rates, whereas P-enrichment would have little to no effect on *C*. *curtipennis* growth. (2) *C*. *curtipennis* would be P-, but not N-, limited, such that only P-fertilization would enhance growth rates. (3) *C*. *curtipennis* experiences N-P co-limitation. In this case, N- or P-fertilization on their own would have little effect, whereas joint N-P fertilization would significantly stimulate *C*. *curtipennis* growth. (4) Increased N-intake by *C*. *curtipennis* will induce P-limitation via altered dietary N:P content. Thus, P-enrichment would have a positive effect on *C*. *curtipennis* growth only under N-enriched conditions.

## Methods

### Study site

We conducted our study during the summer of 2016 at the Konza Prairie Biological Station (KPBS). KPBS is located in the Flint Hills region of northeastern Kansas (USA) and encompasses 3,487 hectares of natural tallgrass prairie. Our experiment was located in an upland, annually burned location protected from both cattle and bison grazing (watershed R1B; see http://kpbs.konza.k-state.edu/treatments.html for a map of KBPS). This site is dominated by C_4_ grasses, chiefly *Andropogon gerardii*, *Sorghastrum nutans*, and *Schizachyrium scoparium*. *Andropogon gerardii*, in particular, can produce over 400 tillers per m^2^ and accounts for over 80% of observed stems at KBPS [42,43]. Consequently, this study focused on the effects of nutrient enrichment of *A*. *gerardii* on insect herbivores. Invertebrate herbivores primarily belong to the orders Cicadellidae, Lepidoptera, Coleoptera, and Orthoptera [6,40]. Among orthopterans, Acrididae are one of the most abundant families, including *C*. *curtipennis* [40]. For all experiments, female *C*. *curtipennis* were collected from watershed R1B in mid-July using a sweep net.

All experiments were performed at KPBS with permission granted by the Konza Prairie LTER.

### N and P enrichment

In early May, we established 24 1 x 1 m plots separated into six blocks. Within a block, four plots were randomly assigned to one of four treatments: control, N-enrichment, P-enrichment, or NP-enrichment. N-enrichment treatments were fertilized with 10 g N m^-2^ in the form of slow-release 46-0-0 urea (CO(NH_2_)_2_, U-Flexx—Koch Agronomic Services LLC). P-enrichment treatments were supplemented with 10 g P m^-2^ contained in 0-18-0 High Yield Super Phosphate (P_2_0_5_, VPG). To verify that our treatments affect plant nutritional content, we collected five leaves of *A*. *gerardii* from each plot at the end of the experiment. Leaves were pooled within each plot, dried at 60°C for 24 hours, and ground for elemental C:N:P analyses at the Soil, Water, and Plant Testing Laboratory at Colorado State University.

### Field experiment

To determine how N- and P-enrichment affect *C*. *curtipennis* performance in natural settings, we used cage enclosure experiments to measure the growth of *C*. *curtipennis* over a five week period from July 9 –August 12. The average maximum temperature during the summer of 2016 at KPBS was 31.67 ± 3.83°C, and the average minimum temperature was 20.22 ± 3.11°C. During this period, relative humidity averaged 68 ± 9%.

Within each 1 x 1 m plot, we constructed a 0.25 x 0.25 x 1.2 m cage (*n* = 6 cages per treatment). Cages were built of four wooden garden stakes covered with fiberglass window screening. The stakes of each cage were driven into the ground, and a skirt of excess screening was attached to the ground using garden staples to prevent grasshopper immigration into or emigration out of the cages during the experiment. On July 9, each cage was stocked with two pre-weighed, female *C*. *curtipennis*. Grasshopper initial weights were 0.08 ± 0.02 (mean ± 1 SE). On August 12, grasshoppers were collected from each enclosure and weighed to obtain a final mass. Individual weights within each cage were averaged to yield one estimate of final mass per replicate. Both the N- and NP-enrichment treatments had two cages in which no grasshoppers were recovered (*n* = 5–6 per treatment).

### Laboratory feeding assays

Concurrent with the field experiment, we conducted laboratory feeding trials to assess the effects of N- and P-enrichment on *C*. *curtipennis* growth in a controlled environment. Forty female *C*. *curtipennis* were placed in individual clear, plastic containers (0.5 L volume) and randomly assigned to one of four treatments (control, N-enrichment, P-enrichment, or NP-enrichment, *n* = 10 per treatment). Containers were kept in a controlled environment exposed to natural, ambient light conditions, allowing grasshoppers to experience a normal circadian rhythm. Temperature was maintained at a 31°:23°C day:night cycle (13:11h), which mimics the natural summer diurnal temperature range at KPBS, using 75 W infrared heat lamps (Exo-Terra). To avoid uneven heating, container positions were randomly shuffled each day. Each container also contained a damp paper towel to replicate humidity in natural conditions.

Feeding trials began on July 15^th^, wherein grasshoppers were fed fresh *A*. *gerardii* collected from the field enrichment plots corresponding to their treatment. Plots varied in their collection date, wherein we randomly chose a plot to sample while ensuring that we did not sample any location more than once. This avoided any systematic sampling of particular blocks which might naturally vary in nutritional quality. Fresh leaves were provided every other day to avoid wilting or other rapid changes in food quality that could potentially affect grasshopper performance. Grasshoppers were weighed every week, including the initial date, until August 12, when the experiment was terminated.

### Data analysis

Plant nutritional content was analyzed with a two-way, blocked MANOVA. This model included main effects, and the interaction between, N and P enrichment as well as a blocking factor. The response was a multivariate matrix that contained the average N and P content of *A*. *gerardii* leaves from each plot.

We analyzed final grasshopper weight in the field experiment using a two-factor, blocked ANOVA that included main and interactive effects for N- and P-enrichment as well as an additive blocking factor. To assess whether N:P imbalance affected *C*. *curtipennis* growth, we calculated the N:P ratio for each plot based on the average foliar N and P values for each plot obtained from analyses of plant nutritional content. We then regressed final *C*. *curtipennis* mass against the N:P ratio.

Laboratory feeding assays were analyzed using a repeated-measures, hierarchical model. Each individual was allowed to have a unique intercept and slope that determined initial mass at Week 0 and weekly growth rate, respectively. The predicted mass at the *i*^th^ week for the *j*^th^ individual was then:

$$\hat{y_{ij}}=\beta_{0j}+\beta_{1j}Week_{ij}$$

where *β*_*0j*_ is the intercept and *β*_*1j*_ is the slope (*i*.*e*. weekly growth rate) for the *j*^th^ individual. The individual intercepts and slopes were multivariate-normally distributed around their respective predictive values. Predicted intercepts and slopes were functions of the nutrient treatment assigned to each individual. For example, the slope (growth rate) of the *j*^th^ individual was:

$$\hat{\beta_{1j}}=\gamma_{0}+\gamma_{1}N_{j}+\gamma_{2}P_{j}+\gamma_{3}N_{j}P_{j}$$

where *N*_*j*_ and *P*_*j*_ are dummy variables denoting whether an individual was fed N- or P-enriched leaves, respectively. This analysis allowed us to determine whether either individual starting mass (*β*_*0j*_) or growth rates (*β*_*1j*_) varied systematically among N- and P-enrichment treatments.

We conducted all analyses in a Bayesian framework to allow us to incorporate prior information into our analyses to avoid the erroneous overestimation of small effect sizes [44]. All response variables were standardized prior to analysis and effects given *N*(0,1) priors, which indicates that we expect most responses to N- or P-enrichment to be within one standard deviation of the response [44] and that large effects should be relatively rare. However, we also present results using uninformative priors, which converge to least-squares estimates, to judge how prior choice influenced our results.

To judge the importance of N- and P- enrichment, we calculated the probability of an effect (Pr) for all parameters, where Pr = 1.00 indicates that a coefficient was entirely positive or negative and Pr = 0.5 indicates that a coefficient was centered around 0 (and therefore not significant) [17]. Generally, Pr = 0.95 is taken as statistically significant, while Pr = 0.90 is taken as moderately significant [17]. Here, we report all probabilities, as well as effect sizes with full uncertainty estimates, to allow the objective assessment of biological importance. Residual analyses for normality and heterogeneous variances indicated that our data met all assumptions for all statistical tests, including the MANOVAs for foliar nutritional content, blocked ANOVAs for field experiments, and the repeated measures regression for laboratory feeding assays. All results given in mean +/- 1 SE unless otherwise stated.

All data are available in S1 File.

## Results

### N and P enrichment

As expected, N-enrichment significantly increase foliar N content in *A*. *gerardii* (Pr = 1.00). *Andropogon gerardii* in control plots contained 0.70 ± 0.04%N in foliar tissues, whereas leaves from N-enriched plants contained 1.10 ± 0.08%N. Phosphorus content was enhanced by P-enrichment (Pr = 0.98), with control leaves possessing 0.06 ± 0.01%P and P-enriched leaves containing 0.12 ± 0.02%P. Neither N- nor P-enrichment affected concentrations of the other element; P-enrichment did not affect foliar N (Pr = 0.37) and N-enrichment did not affect foliar P (Pr = 0.82). Furthermore, there were no significant interactions between N- and P- enrichment for either foliar N or foliar P content (Pr < 0.8 for both interaction terms). The use of less-conservative, uninformative priors did not affect the results.

### Field experiment

**Fig 1:**
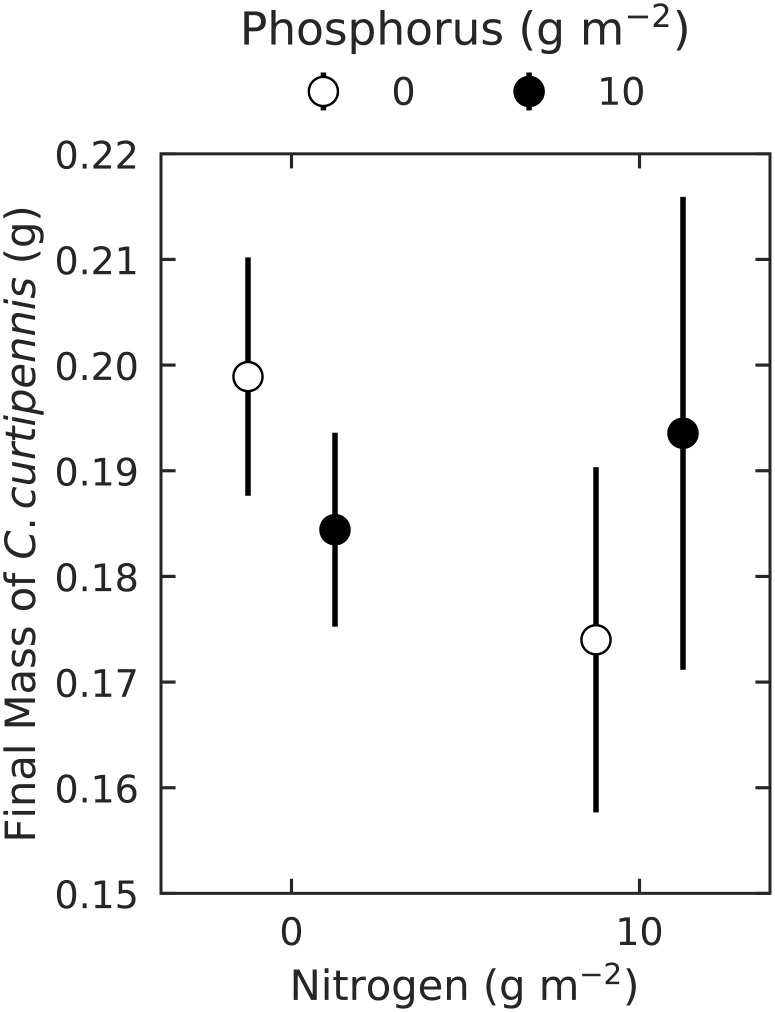
Final mass of *C*. *curtipennis* in all N- and P-enrichment treatments at the end of the field experiment. Points show means ± 1 SE.

None of N-enrichment, nor P-enrichment, or the interaction between N- and P-enrichment affected the final mass of *C*. *curtipennis* (Table 1, Fig 1). However, the use of conservative (*N*(0,1)) priors did influence these results due to our small sample size. Using uninformative priors, there was evidence that P-enrichment reduced grasshopper biomass, but only in the no-nitrogen treatments. Nitrogen enrichment slightly reduced grasshopper mass from 0.19 ± 0.01 g to 0.17 ± 0.02 g without P-enrichment (Pr = 0.87). With added P, N-enrichment had no effect on grasshopper mass (Pr = 0.72). However, such small effect sizes that were not present when using weakly informative priors suggest that neither N- nor P-enrichment strongly affected *C*. *curtipennis* mass in the field experiment. Finally, *C*. *curtipennis* mass was unaffected by the foliar N:P ratio (Pr = 0.57).

**Table 1:** Posterior probabilities for main and interactive effects for N- and P- enrichment treatments in the field experiment.

	N(0,1) Priors	Uninformative Priors
**N-enrichment**	0.76	0.87
**P-enrichment**	0.68	0.80
**N- x P-enrichment**	0.77	0.89

### Laboratory feeding assays

**Fig 2:**
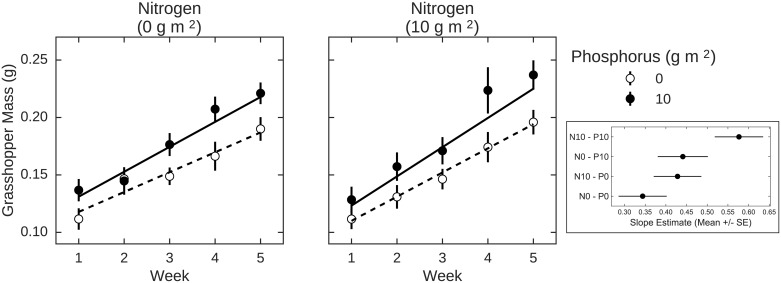
Mass of *C*. *curtipennis* grasshoppers during laboratory feeding assays for all N- and P-enrichment treatments. *Chorthippus curtipennis* mass was measured every week. Points show means ± 1 SE and lines show the best fit for growth rates in each treatment from the model with *N*(0,1) priors. Inset shows the estimated slopes (*i*.*e*. growth rates) for each enrichment treatment.

In laboratory feeding assays, we found weak evidence that both N (Pr = 0.85) and P (Pr = 0.88) increased grasshopper growth rates (Table 2). Grasshoppers grew at an average rate of 0.013 ± 0.003 g week^-1^, whereas grasshoppers fed N-enriched leaves grew 0.017 ± 0.003 g week^-1^, a 30% increase in growth (Pr = 0.85, Fig 2). P-enrichment had a similar effect, enabling grasshoppers to grow at 0.017 ± 0.002 g week^-1^ (Pr = 0.88, Fig 2). We found no evidence of NP co-limitation given the lack of a strong interaction between N- and P-enrichment (Pr = 0.68). The use of uninformative priors did not alter our results (Table 2), suggesting that these patterns, although weak, are robust despite our small sample size.

**Table 2:** Posterior probabilities for main and interactive effects for N- and P- enrichment treatments on individual growth rates in the laboratory feeding assays.

	N(0,1) Priors	Uninformative Priors
**N-enrichment**	0.85	0.82
**P-enrichment**	0.88	0.85
**N- x P-enrichment**	0.68	0.68

## Discussion

Insects partly regulate plant competitive interactions, primary production, and community composition [6,8–12]. The consequences of insect herbivory derive from variable herbivore performance and feeding preferences among plants of differing nutritional content [26]. Nitrogen has long been considered the primary determinant of insect performance [14], although P is also often a limiting nutrient for insect herbivores [29,31,32,45]. Unfortunately, few studies have simultaneously manipulated both N and P in natural field settings to compare the relative strengths of N- and P-limitation and to account for the potential for NP co-limitation of terrestrial herbivores (*e*.*g*. [38,39]). Here, we demonstrate weak evidence for both N and P limitation of *C*. *curtipennis* growth rates in laboratory feeding assays. Importantly, P-limitation was just as strong as N-limitation, but we found no evidence for NP co-limitation in our study.

Insects often perform best when provided N-enriched foods, experiencing higher growth [18–20,46], survival [21–24,47], and reproductive rates [22,25]. Contradicting these results, *C*. *curtipennis* growth rates did not strongly depend on foliar N content in our field experiment. This pattern might arise for several reasons. First, predators exert a strong influence on herbivore foraging behavior [48]. *Chorthippus curtipennis* reduces its foraging effort when exposed to predatory spiders [41]. *Rabidosa rabida* (Lycosidae), the most abundant wolf spider at KPBS, exerts strong top-down control of grasshopper foraging and could potentially negate any response of grasshopper growth to N-enrichment by limiting *C*. *curtipennis* foraging effort [49]. Second, N-fertilization increases plant biomass [26], which can in turn reduce grass canopy light and temperature [47,50]. Since grasshopper foraging is temperature-dependent [51], reductions in temperature caused by fertilization could reduce grasshopper foraging intensity or digestion efficiency, yielding no net impact of fertilization on growth. Thus, the results of field experiments are a complex interaction of predation risk, nutritional quality, and the abiotic environment.

To control for confounding variables, we conducted laboratory feeding assays examining the effects of N-fertilization on *C*. *curtipennis* growth rates. Nitrogen enrichment often enhances growth rates by reducing the stoichiometric mismatch between plants and herbivores [17] and by simultaneously reducing the concentrations of carbon-based defense compounds [52]. Either effect increases plant nutritional quality to herbivores and should yield faster insect growth. In our study, *C*. *curtipennis* growth rates on N-enriched plants increased by ~30% relative to controls. The magnitude of this effect is similar to other studies demonstrating a consistent 20–50% increase of insect growth rates under a similar increase in foliar %N (~0.5% increase in leaf N, [17,39,53]). That our results fall within the range of previously reported effect sizes and are robust to the use of weakly informative priors bolsters our argument that *C*. *curtipennis* experiences N-limited growth at our study site.

Previous studies suggested that P-limitation of insect growth rates is potentially as strong as N-limitation [16]. In support of that that hypothesis, P-enrichment of *A*. *gerardii* resulted in a 30% increase in *C*. *curtipennis* growth rates during laboratory feeding assays, nearly identical in magnitude to that of N-enrichment and within the range of P-enrichment effects seen in other organisms [32,54]. Caterpillars fed P-enriched leaves, for example, were 59% heavier after four weeks [32]. Such increased growth rates occur because higher P-intake rates provide more nutrients for production of ribosomal RNA, a critical component of protein synthesis and somatic growth [29,45,55]. Growth rates can, however, decline if P-intake exceeds a critical threshold [31,32], but the low concentrations of P in natural plant tissues make it unlikely that P-enriched foliage will contain P concentrations that exceed this threshold [16,56]. Furthermore, P-limitation was independent of N-fertilization, suggesting that *C*. *curtipennis* was not co-limited by either N or P, but rather experiences both N- and P-limitation independently.

Most studies assessing the effects of nutrient enrichment on insect performance invoke increased foliar nutrient concentrations as the mechanism for increased plant performance. However, there is a second pathway by which N, and potentially P, enrichment might increased insect growth rates. Herbivore performance declines with increasing physical defenses, many of which are silicon-based [57–59]. Indeed, silicon enrichment stimulates the production of physical defensive structures than can adversely affect the performance of numerous insect herbivores, including orthopterans [57–59], hymenopterans [58], and lepidopterans [60]. However, foliar silicon concentrations are negatively correlated with foliar nitrogen concentrations [58,61]. Nitrogen enrichment might therefore simultaneously increase foliar nutrient concentrations while decreasing physical defense structures [61]. The relative importance of these two mechanisms on insect performance remains an interesting topic for future studies.

It is important to note that all effects of N- and P-enrichment reported here are relatively weak (Tables 1 and 2). Several factors might account for our inability to document strong effects of nutrient enrichment on *C*. *curtipennis* growth rates. First, statistical significance is difficult to achieve with small effect sizes and low replicate numbers (*i*.*e*. *n* = 6 per treatment for the field experiment, *n* = 10 per treatment for the lab experiment). The small sample sizes used here are substantially lower than used in previous studies of either N or P limitation [17,31,32,62,63], and undoubtedly contributed to the lack of significance. Second, methodological issues may have inflated among-individual variation within each treatment in the laboratory trials. We used two infrared heat lamps to maintain a temperature conducive to grasshopper growth, but this method likely created a variable temperature environment. To account for this, we rotated grasshopper positions each day, but since grasshopper growth is temperature-dependent [64], temperature variability among replicates likely hindered our ability to detect strong, significant effects of either N- or P-enrichment on *C*. *curtipennis* growth rates.

Insect growth rates have historically been thought to be primarily N-limited [65]. Recent evidence, however, suggests that P-limitation of insect growth should be as prevalent and as strong as N-limitation [16,56]. Here, we report weak evidence for independent N- and P-limitation of *C*. *curtipennis* growth rates in a tallgrass prairie. Nitrogen-limitation of terrestrial herbivores is expected to increase in strength under future climate scenarios due to more rapid protein denaturing and reduced N-digestion efficiency [17,66]. Phosphorus-limitation also increases in strength under warmed conditions for aquatic herbivores [28] due to higher rRNA:protein ratios, but so far no study has examined how climate change might affect P-limitation of terrestrial insects. Interestingly, nutrient limitation only manifested in controlled laboratory feeding assays and was not evident in field experiments, suggesting that there might be overriding factors that determine insect growth rates in natural environments (*e*.*g*. temperature, predation). Still, our results suggest that P should be jointly considered, along with N, as a primary determinant of herbivore feeding behavior under both current and future climate conditions.

## Supporting information

S1 File A compressed archive containing all data and analytical code presented her. (ZIP)
